# CT and MRI findings of small cell neuroendocrine carcinoma of the urinary bladder: comparison with urothelial carcinoma

**DOI:** 10.1007/s00261-024-04274-z

**Published:** 2024-04-08

**Authors:** Masaya Kawaguchi, Hiroki Kato, Takuya Koie, Yoshifumi Noda, Fuminori Hyodo, Tatsuhiko Miyazaki, Masayuki Matsuo

**Affiliations:** 1 Department of Radiology, Gifu University, 1-1 Yanagido, Gifu, 501-1194 Japan; 2 Department of Radiology, Ogaki Municipal Hospital, 4-86 Minaminokawacho, Ogaki, 503-0864 Japan; 3 Department of Urology, Gifu University, Gifu, Japan; 4 Center for One Medicine Innovative Translational Research (COMIT), Institute for Advanced Study, Gifu University, Gifu, Japan; 5 Department of Pathology, Gifu University, Gifu, Japan

**Keywords:** Small cell carcinoma, Bladder cancer, CT, MRI

## Abstract

**Objective:**

This study aimed to evaluate the efficacy of CT and MRI findings to differentiate small cell neuroendocrine carcinoma (SCNEC) from urothelial carcinoma (UC) of the urinary bladder.

**Materials and methods:**

This study included 90 patients with histopathologically confirmed bladder cancer (10 SCNECs and 80 UCs). Eight patients with bladder SCNEC and 80 with UC underwent CT and MRI, whereas the remaining two patients with SCNEC underwent CT alone before treatment. CT and MRI findings were retrospectively evaluated and compared between the two pathologies.

**Results:**

The maximum diameter (36.5 mm vs. 19.0 mm, *p* < 0.01) and height (22.0 mm vs. 14.0 mm, *p* < 0.01) of the tumor in bladder SCNEC were higher than in UC. The pedunculated configuration (20% vs. 61%, *p* < 0.05) and irregular tumor margins (20% vs. 76%, *p* < 0.01) in bladder SCNEC were less common than in UC. The CT attenuation of the solid component in unenhanced CT images was higher in bladder SCNEC than in UC (37 Hounsfield unit [HU] vs. 34 HU, *p* < 0.01). The apparent diffusion coefficient (ADC) of the solid component in bladder SCNEC was lower than in UC (0.49 × 10^−3^ mm^2^/s vs. 1.02 × 10^−3^ mm^2^/s, *p* < 0.01).

**Conclusion:**

In comparison with UC, bladder SCNEC was larger, had higher unenhanced CT attenuation, and had a lower ADC value. The pedunculated configuration and irregular tumor margins were typical of bladder UC.

**Graphical abstract:**

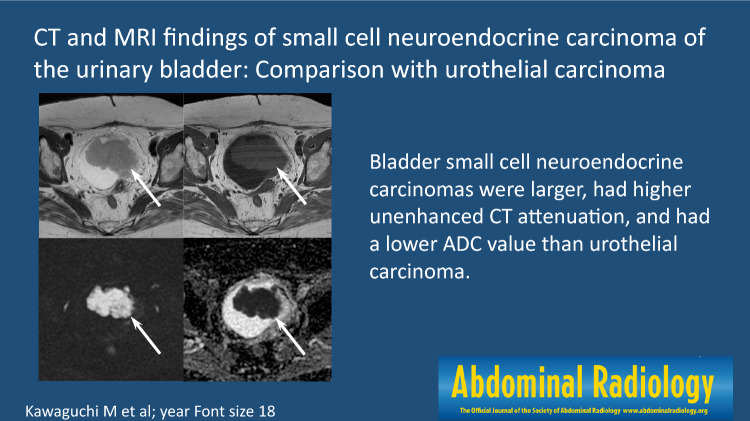

## Introduction

Small cell neuroendocrine carcinoma (SCNEC) is a high-grade tumor of relatively small cells with distinct nuclear characteristics, scant cytoplasm, and neuroendocrine differentiation [1]. Bladder SCNECs are rare histologic variant that account for less than 1% of all bladder malignancies. Bladder SCNEC becomes increasingly prevalent with age, often occurring between the sixth and eighth decades of life (median age, 68 years), and has a male predisposition (a male-to-female ratio of 6:1) [2–4]. The most prevalent symptom of bladder SCNEC is hematuria [2], and cigarette smoking is a risk factor in 50%–70% of cases [4].

Regardless of clinical stage, the National Comprehensive Cancer Network Clinical Practice (NCCN) Guidelines recommend concurrent chemoradiotherapy or neoadjuvant chemotherapy followed by local treatment as standard treatment for patients with bladder SCNEC. Meanwhile, the NCCN Guidelines recommend transurethral resection of bladder tumor (TURBT) alone for non-muscle invasive urothelial carcinoma (UC) and neoadjuvant chemotherapy followed by radical cystectomy or TURBT for muscle invasive UC. Neoadjuvant chemotherapy regimens based on principles of systemic treatment for small cell lung cancer are recommended in treatment of bladder SCNEC [5]; therefore, it is critical to differentiate bladder SCNEC from UC using preoperative imaging for selecting the appropriate treatment.

A large polypoid bladder mass with expansion into the perivesical fat, adjacent organ involvement, and distant metastases was prevalent in a previous study examining CT findings of bladder SCNEC [6]. According to the MRI results, the solid component of the tumor is relatively hypointense-to-isointense to the skeletal muscles on T2-weighted images and shows heterogeneous enhancement [7, 8]. Although a study examining CT imaging characteristics of bladder SCNEC [6, 9] and case reports presenting MRI imaging findings of bladder SCNEC [8, 10] exist, to the best of our knowledge, no study has investigated the CT and MRI differences between bladder SCNEC and UC. Therefore, this study aimed to clarify the CT and MRI findings for differentiating bladder SCNEC from UC.

## Methods

### Patients

This study was approved by the Human Research Committee of our hospital’s institutional review board and the requirement for written informed consent was waived by the board because of the retrospective design. This study was conducted in accordance with the Health Insurance Portability and Accountability Act of 1996. Patients with histopathologically confirmed bladder SCNEC who underwent surgical excision or biopsy at two Japanese institutions were studied from August 2010 to August 2023. We found 250 patients with histopathologically confirmed UC at a single Japanese hospital during the same time period. We randomly selected 80 patients with UC who underwent preoperative CT and MRI because we examined patients with UC ten times as many as bladder SCNEC. This study included 10 patients with bladder SCNEC (age range, 53–86 year; median age, 77 year) and 80 patients with UC (age range, 47–87 year; median age, 72 year). The patient characteristics of bladder SCNEC are shown in Table 1.

**Table 1:** Patient characteristics of bladder SCNEC

	SCNEC (*n* = 10)
*Sex*	
Male	7 (70)
Female	30 (30)
Median age (year) [range]	77 [53–86]
*Symptom*	
Hematuria	8 (80)
Urinary frequency	2 (20)
Urinary retention	1 (10)
Asymptomatic	1 (10)
Smoking history	6 (60)
Tumor marker	
ProGRP (> 80 pg/mL)	5 (50)
NSE (> 16 ng/mL)	1 (11) *n* = 9
*Pathology*	
Pure SCNEC	8 (80)
Mixed SCNEC and UC	2 (20)
Metastasis on initial diagnosis	
Lymph node	2 (20)
Liver	2 (20)
*Treatment*	
Chemotherapy	4 (40)
TURBT + Chemotherapy	3 (30)
Radical cystectomy + Chemotherapy	3 (30)
*Imaging*	
Unenhanced CT	10 (100)
Enhanced CT	7 (70)
Unenhanced MRI	8 (80)

*SCNEC* Small cell neuroendocrine carcinoma, *UC* Urothelial carcinoma, *ProGRP* Pro-gastrin-releasing peptide, *NSE* Neuron-specific enolase, *TURBT* Transurethral resection of bladder tumor. Qualitative data are expressed as raw numbers with percentages in parentheses

### CT Imaging

All patients had CT imaging using an eight-slice CT scanner (LightSpeed Ultra; GE Healthcare, Milwaukee, WI, USA), a 16-slice CT scanner (LightSpeed 16; GE Healthcare, Milwaukee, WI, USA), 64-slice CT scanner (SOMATOM go top; Siemens Healthcare, Erlangen, Germany), or a 64-slice CT scanner (Brilliance CT 64; Philips Healthcare, Best, The Netherlands). All 90 patients had axial unenhanced CT images obtained and 47 patients had axial contrast-enhanced CT images (seven SCNECs and 40 UCs). Contrast-enhanced CT images were obtained 65–100 s after an intravenous injection of 100-mL nonionic iodine contrast material was initiated. Axial and coronal multiplanar reconstruction images were reconstructed with a section thickness ranging from 2.5 to 5 mm and no overlap.

### MRI protocols

MRI was performed using a 1.5-T unit (Intera Achieva 1.5 T Pulsar; Philips Healthcare, Best, The Netherlands), a 1.5-T unit (SIGNA Explorer; GE Healthcare, Milwaukee, WI, USA), a 3.0-T unit (Intera Achieva 3.0 T Quasar Dual; Philips Healthcare, Best, The Netherlands), or 3.0-T unit (DISCOVERY MR750w; GE Healthcare, Milwaukee, WI, USA). All MRI images were obtained with a section thickness of 4–5 mm, an intersection gap of 1 to 2 mm and a field of view of 23 × 23 to 30 × 30 cm. Axial and coronal or sagittal oblique T2-weighted fast spin-echo (TR/TE, 2,586–6,086/90–120 ms), axial T1-weighted spin-echo (TR/TE, 498–789/10 ms), and axial diffusion-weighted single shot spin-echo echo-planar (TR/TE, 4,000–4,800/68–80 ms; b-value = 0 and 1,000 s/mm^2^) images were obtained in 88 patients (eight SCNEC and 80 UC).

### Imaging analysis

All images were independently assessed by two radiologists with 24- and 10-years post-training experience in urogenital imaging, and any disagreements were resolved by consensus. The clinical information and pathological diagnosis were blinded by the reviewers.

First, the maximum diameter and height of the tumor were quantitatively measured. Number (single or multiple), location (dome, right lateral, left lateral, trigone, anterior, or posterior), configuration (pedunculated or broad-based), margins (smooth or irregular), arising in bladder diverticulum, non-neoplastic bladder wall thickening, surrounding fat stranding, lymphadenopathy, and calcification were qualitatively evaluated. If multiple lesions were found, the largest tumor alone was assessed. The acute (≤ 90°) and obtuse (> 90°) angles between the tumor surface and the adjacent bladder wall were used to characterize pedunculated and broad-based lesions, respectively. Irregular margins included spiculated, serrated, and needle-like margins. Arising in bladder diverticulum was defined as a bladder cancer localized within the bladder diverticulum. Non-neoplastic bladder wall thickening was defined as smooth and uniform bladder wall thickening excluding the bladder cancer. Surrounding fat stranding was defined as abnormal increased fat attenuation adjacent to the bladder cancer on CT. A lymph node in the pelvis with a short-axis diameter of more than 8 mm was characterized as lymphadenopathy. Subsequently, CT attenuation (Hounsfield Unit [HU]) of the solid component on unenhanced and contrast-enhanced CT was assessed by positioning the region of interest (ROI) above the tumor.

Second, MRI was used to identify the clinical T category based on the American Joint Committee on Cancer TNM Staging System for Bladder Cancer, eighth edition in 2017. Homogeneity and signal intensity on T1- and T2-weighted images were qualitatively evaluated and signal intensity of the tumor was compared with that of the iliopsoas muscle (low, iso-, or high signal intensity).

Third, the signal intensity ratio on T1- and T2-weighted images and the apparent diffusion coefficient (ADC) value of the solid component were evaluated. A reviewer with 10-year post-training experience in urogenital imaging designated the ROI in the solid component and iliopsoas muscle on the T1- and T2-weighted images and recorded these signal intensities. The ratio of the solid component to the intensity of the muscle signal was computed. ADC values of the solid component were also assessed on ADC maps by positioning ROI on the tumor. ROIs on ADC maps were placed on the solid component as extensively as possible inside the tumor while omitting stalk areas using T2- and contrast-enhanced T1-weighted images.

Finally, the presence and signal intensity of stalk on T2-weighted images and inchworm signs on diffusion-weighted images were evaluated. The signal intensity of the stalk was divided into three categories: low, high, and mixed low and high signal intensity relative to the tumor. The inchworm sign was defined as hyperintense bladder cancer with a hypointense submucosal stalk [11].

### Statistical analysis

All statistical analyses were performed with EZR (Saitama Medical Center, Jichi Medical University, Saitama, Japan), which is a graphical user interface for R (The R Foundation for Statistical Computing, Vienna, Austria). More precisely, it is a modified version of R commander designed to add statistical functions frequently used in biostatistics [12]. The Mann–Whitney *U* test was used to compare quantitative data (age, maximum diameter, height, CT attenuation, signal intensity ratio, and ADC value) between bladder SCNEC and UC. Fisher’s exact test was used to compare the qualitative outcomes (number, location, configuration, margins, arising in bladder diverticulum, non-neoplastic bladder wall thickening, surrounding fat stranding, lymphadenopathy, calcification, clinical T category, homogeneity and signal intensity on T1- and T2-weighted images, stalk, and inchworm sign) between bladder SCNEC and UC. *p* values of < 0.05 were considered significant. *κ* statistics was used to assess the interobserver variability of qualitative assessments. Kappa values of 0.81 to 1.00 exhibit almost perfect agreement; 0.61 to 0.80—substantial agreement; 0.41 to 0.60—moderate agreement; 0.21 to 0.40—fair agreement; and 0.01 to 0.20—slight agreement [13].

## Results

**Fig. 1:**
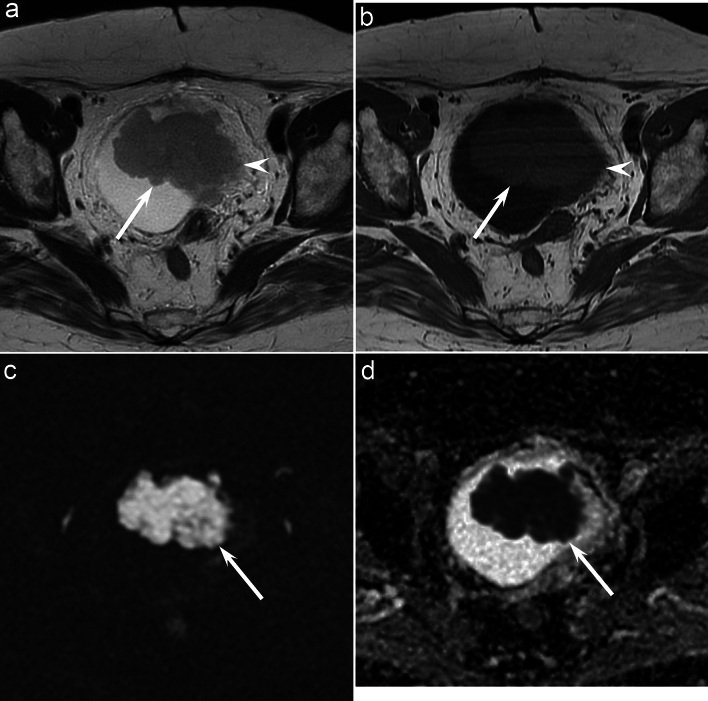
A 76-year-old woman with bladder SCNEC. Axial T2-weighted image **a** and Axial T1-weighted image **b** indicate a broad-based bladder mass (arrow) with a smooth lobulated margin and extension into perivesical fat (arrowhead). **c** Axial diffusion-weighted image shows a mass that is markedly hyperintense (arrow). **d** Axial ADC map shows markedly restricted diffusion with decreased ADC values (0.533 × 10^−3^ mm^2^/s) (arrow)

**Fig. 2:**
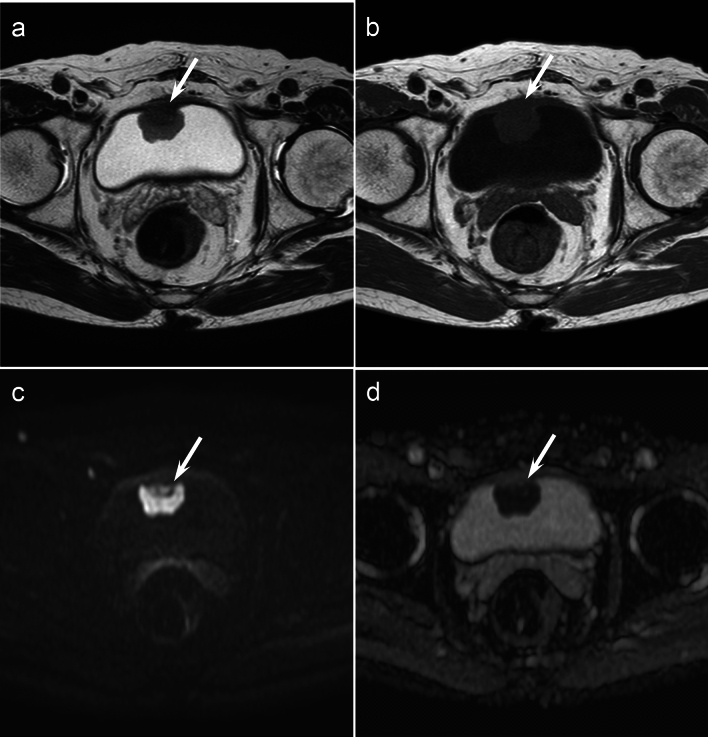
A 77-year-old man with bladder SCNEC. Axial T2-weighted image **a** and Axial T1-weighted image **b** indicate a pedunculated bladder mass (arrow) with a smooth lobulated margin and without extension into perivesical fat. **c** Axial diffusion-weighted image shows a mass that is markedly hyperintense (arrow). **d** Axial ADC map shows markedly restricted diffusion, with decreased ADC values (0.496 × 10^−3^ mm^2^/s) (arrow)

**Fig. 3:**
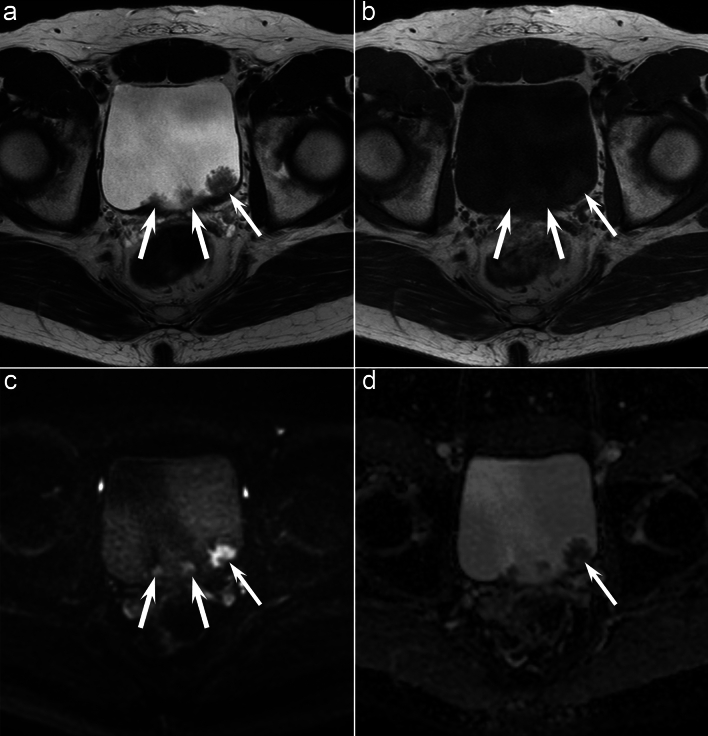
A 54-year-old man with UC. Axial T2-weighted image **a** and axial T1-weighted image **b** showing multiple pedunculated bladder masses (arrows) with irregular margins. **c** Axial diffusion-weighted image shows masses that is hyperintense (arrows). **d** Axial ADC map shows mildly restricted diffusion, with intermediate ADC values (1.376 × 10^−3^ mm^2^/s) (arrow)

The clinical and imaging findings are shown in Table 2. There was no significant difference in gender (*p* = 0.16) and age (*p* = 0.30) between bladder SCNEC and UC. The maximum diameter (36.5 mm vs. 19.0 mm, *p* < 0.01) and height (22.0 mm vs. 14.0 mm, *p* < 0.01) of the tumor in bladder SCNEC were higher than in UC. The pedunculated configuration (20% vs. 61%, *p* < 0.05) and irregular tumor margins (20% vs. 76%,* p* < 0.01) were less common in bladder SCNEC than in UC (Figs. 1, 2, 3). Arising in bladder diverticulum was more prevalent in bladder SCNEC than in UC (20% vs. 1%, *p* < 0.05). On unenhanced CT images, the CT attenuation of the solid component was higher in bladder SCNEC than in UC (37 HU vs. 34 HU, *p* < 0.01). However, there was no significant difference in the number, location, non-neoplastic bladder wall thickening, surrounding fat stranding, lymphadenopathy, calcification, and CT attenuation on contrast-enhanced CT between bladder SCNEC and UC.

**Table 2:** Clinical and imaging findings of bladder SCNEC and UC

	SCNEC (*n* = 10)	UC (*n* = 80)	*p* value	*κ* value
Sex–Male	7 (70)	70 (88)	0.16	–
Age (year)	77 [71–79]	72 [66–79]	0.30	–
Maximum diameter (mm)	37 [32–43]	19 [14–34]	0.005*	–
Height (mm)	22 [17–33]	14 [8–24]	0.012*	–
Number	–	–	0.72	0.64
Single	8 (80)	57 (71)	–	–
Multiple	2 (20)	23 (29)	–	–
Location	–	–	0.21	0.45
Dome	2 (20)	5 (6)	–	–
Right lateral	3 (30)	14 (17)	–	–
Left lateral	2 (20)	21 (26)	–	–
Trigone	1 (10)	29 (36)	–	–
Anterior	1 (10)	6 (7)	–	–
Posterior	1 (10)	5 (6)	–	–
Configuration	–	–	0.018*	0.68
Pedunculated	2 (20)	49 (61)	–	–
Broad-based	8 (80)	21 (39)	–	–
Margins	–	–	< 0.001*	0.44
Smooth	8 (80)	19 (24)	–	–
Irregular	2 (20)	61 (76)	–	–
Arising in bladder diverticulum	2 (20)	1 (1)	0.032*	0.49
Non-neoplastic bladder wall thickening	2 (20)	28 (35)	0.49	0.37
Surrounding fat stranding	0 (0)	1 (1)	> 0.99	0.26
Lymphadenopathy	2 (20)	8 (10)	0.31	0.80
Calcification	2 (20)	18 (23)	> 0.99	0.81
CT attenuation (HU)	–	–	–	–
Unenhanced images	37 [35–48]	34 [27–37]	0.003*	–
Contrast-enhanced images	70 [69–92] (*n* = 7)	76 [65–89] (*n* = 40)	0.89	–

*SCNEC* Small cell neuroendocrine carcinoma, *UC* Urothelial carcinoma, *HU* Hounsfield Unit. Quantitative data are expressed as medians with interquartile in square brackets. Qualitative data are expressed as raw numbers with percentages in parentheses.

*Significant differences were observed between bladder SCNEC and UC (*p* < 0.05)

MRI findings are shown in Table 3. The ADC value of the solid component in bladder SCNEC was lower than in UC (0.49 vs. 1.02 × 10^−3^ mm^2^/s, *p* < 0.01) (Figs. 1, 2, 3). However, there was no significant difference between bladder SCNEC and UC in the clinical T category, homogeneity and signal intensity on T1-weighted images, homogeneity and signal intensity on T2-weighted images, and signal intensity ratio on T1- and T2- weighted images. Table 4 summarizes the differences in imaging findings of bladder SCNEC and UC.

**Table 3:** MRI findings of bladder SCNEC and UC

	SCNEC (*n* = 8)	UC (*n* = 80)	*p* value	*κ* value
Clinical T category	–	–	0.07	0.72
1	3 (38)	58 (72)	–	–
2	3 (38)	8 (10)	–	–
3	3 (38)	8 (10)	–	–
4	1 (12)	7 (9)	–	–
T1-weighted images	–	–	–	–
Homogeneity	–	–	> 0.99	0.28
Homogeneous	8 (100)	76 (95)	–	–
Heterogeneous	0 (0)	4 (5)	–	–
Signal intensity	–	–	–	–
Low signal intensity	1 (13)	1 (1)	0.18	0.40
Iso-signal intensity	7 (87)	79 (99)	–	–
T2-weighted images	–	–	–	–
Homogeneity	–	–	–	–
Homogeneous	6 (75)	55 (69)	> 0.99	0.27
Heterogeneous	2 (25)	25 (31)	–	–
Signal intensity	–	–	–	–
High signal intensity	8 (100)	80 (100)	> 0.99	1.00
Signal intensity ratio	–	–	–	–
T1-weighted images	1.19 [1.07–1.30]	1.23 [1.10–1.32]	0.83	–
T2-weighted images	4.23 [3.36–6.46]	5.73 [4.45–7.74]	0.07	–
ADC value (× 10^−3^ mm^2^/s)	0.49 [0.48–0.50]	1.02 [0.86–1.16]	< 0.001*	–
Stalk on T2-weighted images	–	–	–	0.60
Presence	2 (25)	47 (59)	0.14	–
Signal intensity of stalk	–	–	0.32	–
Low signal intensity	1 (50)	10 (21)	–	–
High signal intensity	0 (0)	21 (45)	–	–
Mixed low and high signal intensity	1 (50)	16 (34)	–	–
Inchworm sign on diffusion-weighted images	2 (25)	50 (63)	0.06	0.61

*SCNEC* Small cell neuroendocrine carcinoma, *UC* Urothelial carcinoma, *ADC* Apparent diffusion coefficient. Quantitative data are expressed as medians with interquartile in square brackets. Qualitative data are expressed as raw numbers with percentages in parentheses.

*Significant difference was observed in between SCNEC and UC (*p* < 0.05)

**Table 4:** Summary of differences in imaging findings of bladder SCNEC and UC

	SCNEC	UC	*p* value
Maximum diameter (mm)	37	19	0.005*
Height (mm)	22	14	0.012*
Broad-based configuration	80%	39%	0.018*
Smooth margins	80%	24%	< 0.001*
CT attenuation on unenhanced images (HU)	37	34	0.003*
ADC value (× 10^−3^ mm^2^/s)	0.49	1.02	< 0.001*

*SCNEC* Small cell neuroendocrine carcinoma, *UC* Urothelial carcinoma, *ADC* Apparent diffusion coefficient.

*Significant differences were observed between SCNEC and UC (*p* < 0.05)

The stalk was observed on T2-weighted images in 25% of bladder SCNECs and 59% of UCs. Stalk signal intensities on T2-weighted images were low, high, and mixed low and high in 50%, 0%, and 50% of bladder SCNECs and 21%, 45%, and 34% of UCs, respectively. Hyperintense stalk on T2-weighted images was observed in UCs alone. There was no significant difference in inchworm signs between bladder SCNEC and UC.

The two observers’ *κ* values showed fair agreement for the number, non-neoplastic bladder wall thickening, signal intensity on T1-weighted images, and　homogeneity on T1- and T2-weighted images and moderate agreement for the location, margins, arising in bladder diverticulum, and stalk. Regarding the other findings, there was a substantial or almost perfect agreement.

## Discussion

In this study, the maximum diameter and height of the tumor were larger in bladder SCNEC than in UC. The pedunculated configuration and irregular tumor margins were more common in UC than in bladder SCNEC. On unenhanced CT images, the CT attenuation of the solid component in bladder SCNEC was higher than in UC. The ADC value of the solid component in bladder SCNEC was lower than in UC. Hyperintense stalk on T2-weighted images was observed in UC alone.

In the present study, the bladder SCNEC was significantly larger than UC. Previous studies and case reports indicate that the average maximum diameter of the tumor was 5.05 cm (range 1.5–13 cm) [6, 8, 10, 14, 15]. In contrast, the average size of UC was 2.1–3.3 cm, and UCs larger than 3 cm had a high recurrence rate or were highly aggressive [16–18]. Therefore, large tumor size is an important finding in suggesting bladder SCNEC.

In this study, the pedunculated configuration was more common in UC than in bladder SCNEC, whereas the advanced clinical T category was more common in bladder SCNEC than in UC. Papillary or pedunculated bladder cancer with a stalk has a better prognosis, and papillary or pedunculated configuration is typically classified as the T1 category [11, 19]. Previous studies of 11 bladder SCNECs with available CT or MRI findings revealed that the configuration was pedunculated in one case and broad-based in the remaining 10 cases, and nine (82%) of 11 cases were locally advanced tumors (T3 or T4) [6, 8, 10, 15]. Although broad-based configuration may help diagnose bladder SCNEC, it can also be observed in locally advanced UC.

The present study found that irregular margins were more common in UC than in bladder SCNEC. UCs exhibit a variety of gross appearances, including papillary, sessile, polypoid, nodular, and ulcerative morphology [20]. This variety of gross findings might lead to UC tumor margin irregularities. In contrast, bladder SCNECs often have a diffuse, sheet-like morphology with no papillary structure [1].

In this study, bladder SCNEC had higher CT attenuation of the solid component on unenhanced CT than UC, and the ADC value was lower in bladder SCNEC than in UC. In general, high CT attenuation of the solid component indicates high cellularity [21, 22]. SCNEC, a hypercellular tumor, is classified as a small round cell tumor because it comprises dense sheets of small cells with a high nucleus-to-cytoplasm ratio and is densely packed with scant cytoplasm [1, 23]. Previous studies found that the ADC values of SCNEC in the uterus and paranasal sinus were considerably low (0.64–0.70 × 10^−3^ mm^2^/s) [24, 25], which is consistent with our findings. Although UC had relatively high ADC values (0.73–1.28 × 10^−3^ mm^2^/s) [16] [17, 18, 26–28], high-grade or highly aggressive UC tended to show low ADC values (0.73–0.79 × 10^−3^ mm^2^/s) [16, 18, 27, 28]. However, in this study, the ADC values of bladder SCNEC were lower than those of high-grade or highly aggressive UC in previous studies. Thus, the ADC value of the solid component would be a reliable finding for differentiating bladder SCNEC from UC.

The present study found hyperintense stalk on T2-weighted images in UC alone. The signal intensity of the stalks of UC on T2-weighted images was reported to be low, high, and mixed low and high at 82%, 9%, and 9%, respectively [29]. The signal intensity of the stalk on T2-weighted image changes with fibrous and edematous stroma ratio [29]. The absence of hyperintense stalk on T2-weighted images in bladder SCNEC may be due to a lack of edematous stroma, and hyperintense stalk on T2-weighted images would be a reliable finding for diagnosing UC.

The present study revealed considerable differences of CT and MRI findings between bladder SCNEC and UC. Bladder SCNECs require distant metastatic survey and systemic treatment based on small cell carcinoma of the lung [5]. If radiologists can make a diagnosis of bladder SCNEC using CT or MRI, a prompt survey for distant metastases including brain and bone metastases can be achieved prior pathological diagnosis, which can avoid delay in the initiation of appropriate systemic treatment.

This study has several limitations. First, due to the rarity of bladder SCNEC, this study only included a few patients. Further investigation with increasing the sample size is required to validate the reliability of CT and MRI in distinguishing bladder SCNEC from UC. Second, contrast-enhanced MRI was not evaluated because only two patients with bladder SCNEC had it done. Third, diffusion-weighted images were obtained using MRI devices ranging from 1.5 to 3 T. Finally, this study did not investigate the availability of radiomics or artificial intelligence; however, the utility of them has been reported in terms of accurate diagnosis, muscle invasion, and personalized treatment of bladder cancer [30, 31]. These advanced imaging technologies are expected to be applicable to differentiate between bladder SCNEC and UC.

In conclusion, bladder SCNEC was larger, had higher unenhanced CT attenuation, and had a lower ADC value than UC. Conversely, pedunculated configuration and irregular tumor margins were characteristic configurations of UC. These imaging findings can help differentiate bladder SCNEC from UC. Accurate diagnosis of bladder SCNEC using CT and MRI leads to prompt initiation of appropriate systemic treatment without delay in decision of clinical staging.
